# Postoperative trachomatous trichiasis: a systematic review and meta-analysis study

**DOI:** 10.1093/inthealth/ihad014

**Published:** 2023-02-28

**Authors:** Nebiyat Feleke Adimassu, Abel Sinshaw Assem, Sofonias Addis Fekadu

**Affiliations:** Department of Optometry, School of Medicine, College of Medicine and Health Sciences, University of Gondar, Gondar, Ethiopia; Department of Optometry, School of Medicine, College of Medicine and Health Sciences, University of Gondar, Gondar, Ethiopia; Department of Optometry, School of Medicine, College of Medicine and Health Sciences, University of Gondar, Gondar, Ethiopia

**Keywords:** postoperative, recurrence, surgery, trachoma, trichiasis

## Abstract

**Background:**

Trachomatous trichiasis is the potential stage of trachoma in which the eyelashes scratch the surface of the globe, ultimately causing corneal opacity, visual impairment and blindness. The aim of this systematic review and meta-analysis is to obtain the pooled prevalence and associated factors of postoperative trachomatous trichiasis (PTT) in World Health Organization (WHO) trachoma-endemic regions.

**Methods:**

An inclusive literature search was undertaken using PubMed, Cochrane Library, Science Direct and Google Scholar databases from 30 May 2022 to 28 June 2022. I^2^ statistics and funnel plots were used to determine heterogeneity and publication bias among included studies. A random effects model was used to estimate pooled prevalence, incidence and odds ratios (ORs) with the respective 95% confidence intervals (CIs) using RevMan 5.4 software.

**Results:**

Eighteen articles were included in this meta-analysis and systematic review. The pooled prevalence of PTT was 19% (range 18–21). PTT was lower among young adults compared with old adults (OR 0.63 [95% CI 0.44 to 0.92]), single-dose oral azithromycin as compared with tetracycline eye ointment users (OR 0.82 [95% CI 0.69 to 0.99]) and minor trichiasis before surgery as compared with major trichiasis (OR 0.63 [95% CI 0.47 to 0.85]).

**Conclusions:**

The incidence of PTT was higher than the WHO’s recommendation. Prescribing single-dose oral azithromycin after surgery, periodic training for trichiasis surgeons, close follow-up and health education after surgery are crucial to minimize the recurrence.

Study protocol registration on PROSPERO: CRD42022336003

## Introduction

Trachoma is caused by an intracellular bacterial infection with *Chlamydia trachomatis* and is among the leading causes of preventable blindness globally.^1,2^ Trachomatous trichiasis is the presence of any lash that touches the globe as a consequence of progressive conjunctival scarring caused by repeated infection with *C. trachomatis*.^3,4^ Trachomatous trichiasis is the potential stage of trachoma in which the eyelashes scratch the surface of the globe, ultimately causing corneal opacity, visual impairment and blindness.^5,6^ The World Health Assembly declared a resolution calling for the elimination of blinding trachoma as a public health problem by 2020.^7^ According to current estimates, 2 million people require trachomatous trichiasis surgery.^8^ The World Health Organization (WHO) recommends the SAFE (surgery, antibiotics, facial cleanliness, environmental improvement) strategy for the control of trachoma.^9^ Surgery for trichiasis is one of the pillars of the WHO's strategy for the global elimination of trachoma as a public health problem.^7^ Trichiasis surgery repositions the eyelid by externally rotating it to prevent the eyelashes from touching the eyeball.^10^ Even though there are various options for trichiasis surgery, the most widely used procedures, and those advocated by the WHO, are bilamellar tarsal rotation (BLTR) and posterior lamellar tarsal rotation (PLTR).^11^

Unfavourable surgical outcomes like postoperative trachomatous trichiasis (PTT) play a role in increasing trachoma-related blindness and cause other trichiasis patients to decline surgery. PTT should be emphasised because reoperation has a higher risk of surgical failure than the primary operation, increasing the risk of blindness.^12^ The risk factors for PTT include the severity of trichiasis at baseline, conjunctival inflammation at the follow-up, female gender and a high-risk area of residence.^13–15^ Overall, PTT is related in part to the surgical skill of the surgeon.^16^ Trichiasis surgery substantially improves both vision-related quality of life and health-related quality of life regardless of visual acuity changes.^17^

According to the WHO, the prevalence of PTT should be ≤10% at the 1-y follow-up.^7^ However, several studies have reported PTT ranging from 2.30% to 50.60% within 1 y, and longer-term recurrence rates are considerably higher than this. Various studies have been conducted throughout the world regarding PTT and its associated factors. This systematic review aimed to obtain the pooled prevalence and associated factors of PTT in WHO trachoma-endemic regions.

## Methods

### Definitions

PTT is defined as evidence of any misdirected lash that touches the globe (eyeball) after trichiasis surgery. The type of surgery considered in this study was incisional surgery; laser electrolysis and cryotherapy were not included.

Baseline trichiasis was categorized as major and minor. Major trichiasis is defined as five or more that touch the globe at baseline. Participants ≤60 y of age were grouped into the young age group and those ≥60 y of age were grouped into the old age category.

### Search strategy

This review was conducted based on the Preferred Reporting Items for Systematic Reviews and Meta-Analyses (PRISMA) statement guidelines.^18^ The study protocol was registered on PROSPERO (CRD42022336003).

An inclusive literature search was undertaken using PubMed, Cochrane Library, Science Direct and Google Scholar databases from 1 to 30 August 2021. The search strategy used the following MeSH terms: [‘Trichiasis’ OR ‘post-operative trichiasis’ OR ‘post-surgical trichiasis’ OR ‘trichiasis recurrence’ OR ‘trichiasis surgical outcome’].

Duplicate entries and the citation process were managed by Endnote version 7 reference manager software (Thomson Reuters, London, UK).

### Study selection

Two reviewers (NF and SA) independently screened the titles and abstracts of the articles found to determine their potential eligibility for inclusion. The full texts of potentially eligible studies were obtained. Selection for inclusion into the review was conducted by the two reviewers working independently. Any disagreements regarding inclusion of studies were resolved by discussion or by consulting the third reviewer (AS).

### Inclusion and exclusion criteria

All original observational and interventional research in WHO trachoma-endemic regions that were satisfied the following criteria were included: published in the English language after 2000, measured PTT (defined as one or more eyelashes touching the eyeball or evidence of epilation of in-turned eyelashes after surgery) as an outcome, included participants who had trachomatous trichiasis in at least one eye and included incisional trachomatous trichiasis surgery. Editorial articles, reviews, expert opinion pieces, conference papers and meeting abstracts; studies that did not have an incisional surgical intervention; those that did not measure or report PTT as an outcome; review articles, conference abstracts and proceedings, editorials and case reports and studies without full-text access were excluded.

### Outcome of interest

The primary outcome of interest was the pooled prevalence and incidence of PTT. Moreover, the results were stratified by age group (old versus adult), baseline trichiasis (major versus minor) and type of antibiotic used after surgery (tetracycline eye ointment versus single-dose oral azithromycin).

### Data extraction and risk of bias assessment

Data were extracted from the studies using Excel (Microsoft, Redmond, WA, USA). The following parameters were extracted from each included study: author and year of publication, study design, study period, duration of follow-up and type of surgery. Trichiasis recurrence was also extracted in terms of the following categories: young versus old, major versus minor trichiasis at baseline and tetracycline versus single-dose oral azithromycin use after surgery.

The methodological quality of the articles included in this review was assessed using the Joanna Briggs Institute's (JBI) critical appraisal checklist for randomized clinical trials, prevalence and cohort studies.^19^ Based on the JBI checklist, all studies involved in this analysis had a quality score >50%. Critical appraisal was conducted independently by two reviewers, with disagreements resolved through discussion or by consulting the third reviewer.

### Statistical analysis

The extracted data were exported and entered in RevMan 5.4 for quantitative analysis. The random effects model (DerSimonian–Laird method) was used for prevalence analysis and odds ratio (OR) with the respective 95% confidence intervals (CIs).

Studies included in this systematic review were checked for heterogeneity and risk of bias. Statistical heterogeneity was assessed using I^2^ and χ^2^) tests. I^2^ values of 25%, 50% and 75% were considered as low, medium and high heterogeneity, respectively. The robustness of the pooled estimate and the impact of a single study on aggregate result was determined by sensitivity analysis using funnel plots.

## Results

### Characteristics of included studies

**Figure 1.**
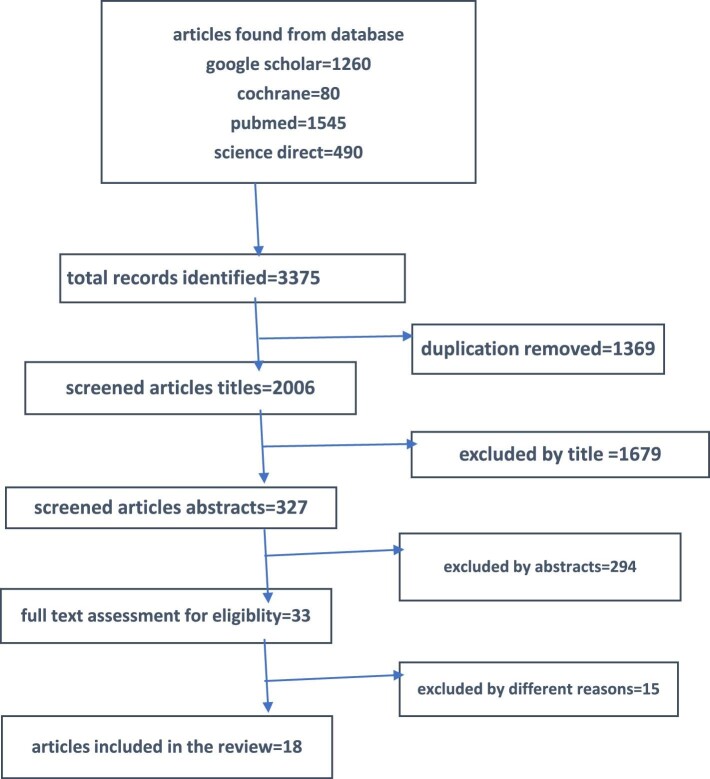
PRISMA flow guide for PTT recurrence.

Initially, through electronic search, 3375 articles were found. After reviewing those articles for duplication, 2006 articles were selected for review by title and abstract. After deep review, 18 articles were included in the study (Figure 1). The included studies are summarized in Table 1.

**Table 1.** Summary of included studies for systematic review and meta-analysis of PTT

First author, year	Country	Study design	Eyelids, n	Type of surgery	Intervention	Length of follow-up
Ahmed, 2015	Egypt	Cross-sectional	752	ALR	–	6 months
Assefa, 2008	Ethiopia	Cross-sectional	560	PLTR	–	1 y
Burton, 2005	Gambia	RCT	426	PLTR	Preoperative azithromycin	1 y
Gower, 2011	Ethiopia	RCT	2601	BLTR	Preoperative azithromycin	6 weeks
Habtamu, 2017	Ethiopia	RCT	1000	BLTR and PLTR	PLTR versus BLTR	1 year
Khandakar, 2001	Oman	Cohort	293	PLTR	–	1.5–4 y
Khandakar, 2009	Vietnam	Interventional	648	Modified Cuenod Nataf lid surgery	1 versus 2 y	1 y and 2 y
Merbs, 2015	Tanzania	Cohort	630	BLTR	–	Minimum of 18 months
Pearson, 2013	Ethiopia	Cross sectional	363	BLTR	–	11 months
Rajak, 2010	Gambia	Prospective cross-sectional	356	PLTR	–	4 y
Rajak, 2013	Ethiopia	Prospective cross-sectional	1300	PLTR	–	2 y
Ramily, 2016	Nigeria	Cohort	133	Lid surgery	–	6 months
Thanh, 2004	Vietnam	Prospective	263	Modified Cuenod Nataf lid surgery	–	1 y
West, 2005	Tanzania	Cohort	384	Not specified	–	>18 months
West, 2006	Ethiopia	RCT	1452	Not specified	Single-dose azithromycin	1 y
Woreta, 2011	Ethiopia	Prospective cross-sectional	488	BLTR	–	6 months
Zhang, 2004	Nepal	Cohort	79	BLTR	–	12 months
Zhang, 2006	Nepal	Case–control	109	BLTR	Azithromycin versus placebo	1 y

ALR: anterior lamellar reposition; RCT: randomized controlled trial.

### PTT recurrence rate

**Figure 2.**
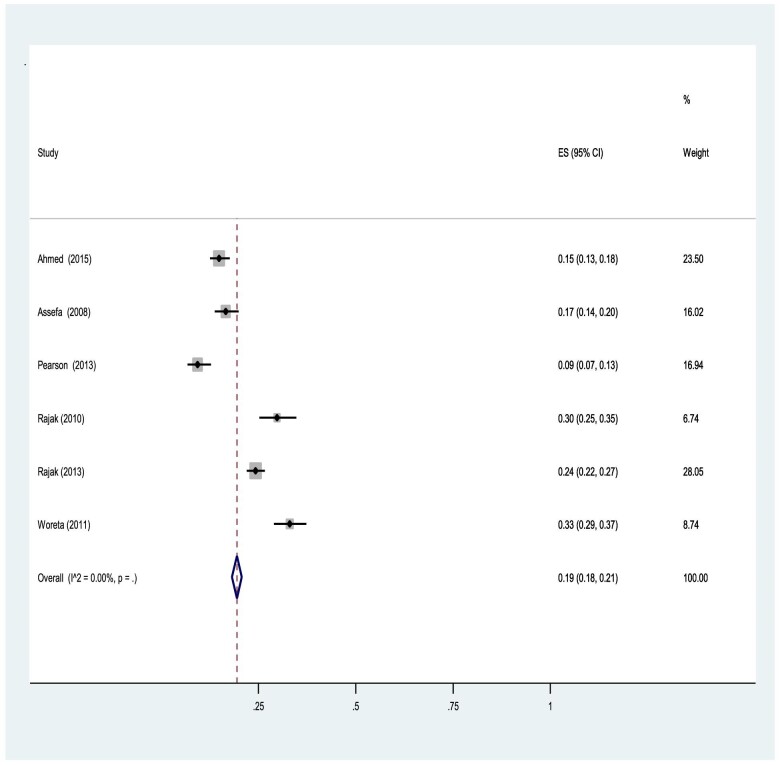
Meta-analysis showing pooled prevalence for PTT.

Original articles regarding PTT were reviewed across trachoma-endemic countries of the world. The pooled prevalence and incidence of PTT was 19% (range 18–21) (Figure 2). The outcome was reviewed and analysed in different subgroups, including age of the participant, type of antibiotics used after surgery and trichiasis severity at baseline.

### Single-dose oral azithromycin versus tetracycline eye ointment

We reviewed four studies regarding the effect of single-dose azithromycin versus tetracycline treatment after trichiasis surgery. A randomized controlled trial was done in Ethiopia in 2006 to compare azithromycin and topical tetracycline in the reduction of PTT. A single dose of postsurgical azithromycin was significantly associated with a 33% reduction in PTT up to 1 y compared with topical tetracycline prescribed for 6 weeks.^20^ Patients randomized to receive azithromycin had significantly fewer severe recurrences (4.2/100 person-years) compared with those randomized to topical tetracycline (7.9/100 person-years). A single dose of azithromycin has been shown to reduce severe PTT.^21^ Another study was done in Ethiopia in 2011 to determine whether treatment with oral azithromycin or topical tetracycline reduces PTT. Even though it was not statistically significant, the prevalence of PTT was 10% in the azithromycin group and 13% in the tetracycline group. The azithromycin group had a 22% reduction in PTT.^22^

A randomized controlled trial in Gambia reported that there was no difference in PTT between the azithromycin and tetracycline groups.^5^

A study from Nepal reported by Zhang et al.^23^ suggested that azithromycin treatment at the time of surgery reduced PTT as compared with placebo.

**Figure 3.**
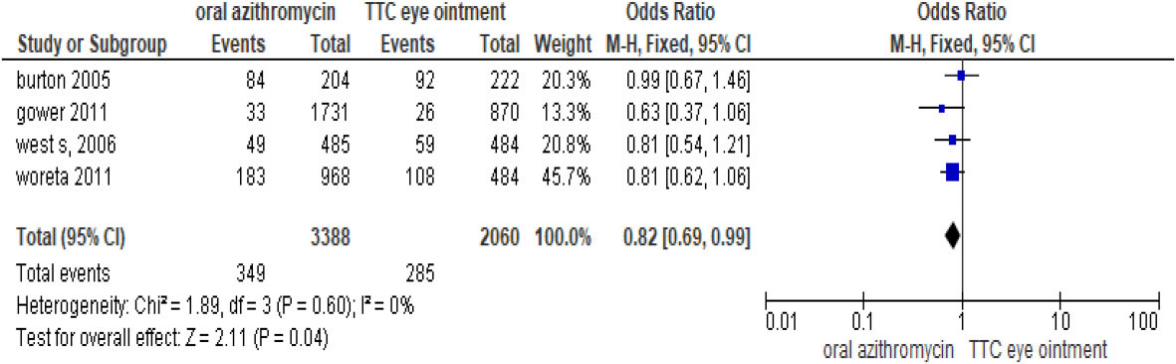
Meta-analysis output comparing single-dose oral azithromycin and tetracycline eye ointment for PTT.

According to this meta-analysis, taking oral azithromycin after surgery minimized the risk of PTT compared with tetracycline eye ointment (OR 0.82 [95% CI 0.69 to 0.99]) (Figure 3).

### Age

**Figure 4.**
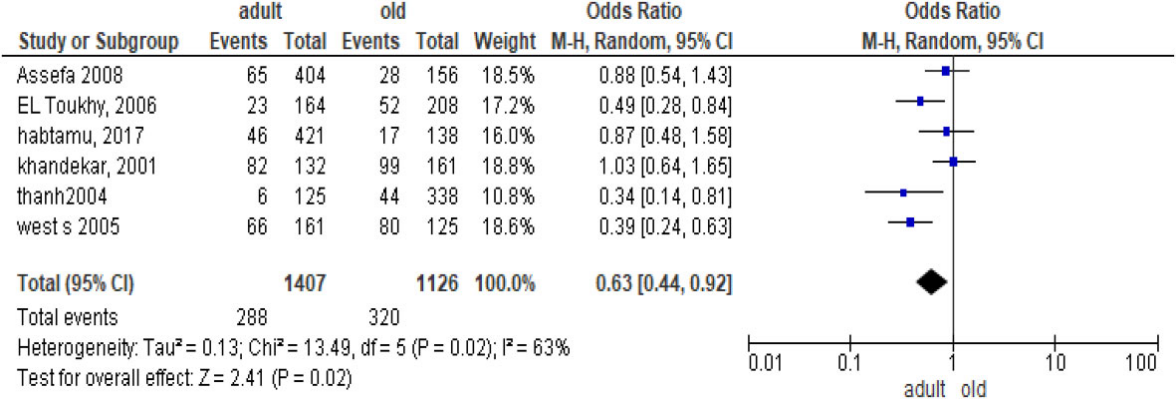
Meta-analysis output comparing old adults and young adults for PTT.

Six studies reported the effect of age on PTT. According to these different observational and interventional studies, PTT was higher among old age groups as compared with young age groups.^13,24,25,27^ This meta-analysis also supported that adult age groups were less likely to develop PTT (OR 0.63 [95% CI 0.44 to 0.92]) (Figure 4).

### Severity of baseline trichiasis (major versus minor)

**Figure 5.**
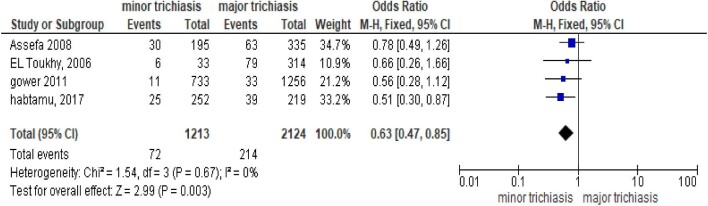
Meta-analysis output comparing severity of trichiasis at baseline for PTT.

Four different studies reported the effect of severity of baseline trichiasis on PTT. The studies concluded that PTT was higher among participants with major trichiasis before surgery.^4,11,16^ According to the meta-analysis of those studies, patients with major trichiasis at baseline had a higher probability of developing PTT as compared with those who had minor trichiasis (OR 0.63 [95% CI 0.47 to 0.85]) (Figure 5).

## Discussion

According to WHO recommendation, the prevalence of PTT should be <10%.^7^ However, the pooled prevalence of PTT in this meta-analysis is higher than the recommended. Hence, investigating and intervening in factors contributing to a high recurrence rate is crucial to eliminate PTT.

After a thorough review of included studies, we found that PTT has an association with different factors. Age is one factor that contributes to PTT. Even if there was no experimental study done to compare old adults with young adults, different observational studies have shown that older adults have a tendency towards PTT.^5,11,15,24^ This meta-analysis also found that PTT was higher in old adults (26.22%) compared with young adults (17.42%). The reason for higher PTT among old adults could be because of involutional changes, as the skin and tarsal plate of the eyelid become atrophied, increasing the laxity of the eyelid. When the pre-tarsal orbicularis muscle is comparatively strong, the eyelid may become inverted and develop entropion.^28^

PTT was higher among females as compared with males.^21,25,29^ The reason could be women spend more time with children than men and young children are the reservoirs of trachoma infection. Having contact with them may cause more frequent trachoma infections after surgery.

Those participants with major trichiasis prior to the surgery had a higher prevalence of recurrent trichiasis.^4,5,11,16,30^ In this meta-analysis, the prevalence of PTT among participants with major trichiasis at baseline was 10.66%, whereas in those with minor trichiasis it was 6.66%. Studies showed peripheral trichiasis at baseline was a risk factor for recurrence. Major trichiasis with a peripheral location increases the recurrence of trichiasis after lid surgery.^30^

According to randomized clinical trials, single-dose oral azithromycin reduces the recurrence rate of trichiasis as compared with tetracycline eye ointment.^20–23^ This meta-analysis also showed that prescribing single-dose oral azithromycin after surgery had the potential to reduce the risk of PTT. The reason may be because the efficacy of azithromycin for *Chlamydia* infection is better than tetracycline. The ease of administration for single-dose azithromycin increases the compliance as compared with longer-duration tetracycline eye ointment, which ultimately reduces PTT.

PTT was also increased by the presence of active conjunctival infection and inflammation, including active trachoma. Also, residents in high-risk areas for trachoma reinfection demonstrated high PTT.^13,14,15,25^ The finding could be because conjunctival infection and inflammation lead to conjunctival scar, which eventually forms entropion and trichiasis.

The surgeon’s skill was repeatedly associated with trichiasis recurrence.^11,15,16,26^ This may be because surgeons with poor skill and experience produce an irregular and inadequate dissection of the incision. Incision irregularity and poor dissection of the incision increase the risk of PTT.^4,16^

## Conclusions

In conclusion, the prevalence of PTT was higher than the WHO's recommendation. Prescribing single-dose oral azithromycin, periodic training for trichiasis surgeons, close follow-up and health education after surgery are crucial to minimize PTT.

## Data Availability

All required data are included in the article.
